# Interleukin 1 beta-induced chloride currents are important in osteoarthritis onset: an in vitro study

**DOI:** 10.1093/abbs/gmab010

**Published:** 2021-03-02

**Authors:** Zhiqin Deng, Zicong Lin, Qing Zhong, Minqiang Lu, Huankun Fang, Jianquan Liu, Li Duan, Lixin Chen, Liwei Wang, Daping Wang, Wencui Li

**Affiliations:** Guangdong Provincial Research Center for Artificial Intelligence and Digital Orthopedic Technology, Hand and Foot Surgery Department, Shenzhen Second People’s Hospital (The First Hospital Affiliated to Shenzhen University), Shenzhen 518000, China; Guangdong Provincial Research Center for Artificial Intelligence and Digital Orthopedic Technology, Hand and Foot Surgery Department, Shenzhen Second People’s Hospital (The First Hospital Affiliated to Shenzhen University), Shenzhen 518000, China; Guangdong Provincial Research Center for Artificial Intelligence and Digital Orthopedic Technology, Hand and Foot Surgery Department, Shenzhen Second People’s Hospital (The First Hospital Affiliated to Shenzhen University), Shenzhen 518000, China; Guangdong Provincial Research Center for Artificial Intelligence and Digital Orthopedic Technology, Hand and Foot Surgery Department, Shenzhen Second People’s Hospital (The First Hospital Affiliated to Shenzhen University), Shenzhen 518000, China; Guangdong Provincial Research Center for Artificial Intelligence and Digital Orthopedic Technology, Hand and Foot Surgery Department, Shenzhen Second People’s Hospital (The First Hospital Affiliated to Shenzhen University), Shenzhen 518000, China; Guangdong Provincial Research Center for Artificial Intelligence and Digital Orthopedic Technology, Hand and Foot Surgery Department, Shenzhen Second People’s Hospital (The First Hospital Affiliated to Shenzhen University), Shenzhen 518000, China; Guangdong Provincial Research Center for Artificial Intelligence and Digital Orthopedic Technology, Hand and Foot Surgery Department, Shenzhen Second People’s Hospital (The First Hospital Affiliated to Shenzhen University), Shenzhen 518000, China; Department of Pharmacology, Medical College, Jinan University, Guangzhou 510632, China; Department of Physiology, Medical College, Jinan University, Guangzhou 510632, China; International School, Jinan University, Guangzhou 510632, China; Guangdong Provincial Research Center for Artificial Intelligence and Digital Orthopedic Technology, Hand and Foot Surgery Department, Shenzhen Second People’s Hospital (The First Hospital Affiliated to Shenzhen University), Shenzhen 518000, China; Guangdong Provincial Research Center for Artificial Intelligence and Digital Orthopedic Technology, Hand and Foot Surgery Department, Shenzhen Second People’s Hospital (The First Hospital Affiliated to Shenzhen University), Shenzhen 518000, China

**Keywords:** hypotonic, chloride channel, IL-1β, osteoarthritis, inflammation

## Abstract

Persistent hypotonic and inflammatory conditions in the joint cavity can lead to the loss of cartilage matrix and cell death, which are the important mechanisms of osteoarthritis (OA) onset. Previous studies have confirmed that the existence of a hypotonic environment is a red flag for inflammation, as hypotonic environment induces the opening of the chloride channel of the cell and promotes chloride ion efflux, which prompts the cell volume to increase. Chloride channels play an important role in the regulation of mineralization and chondrocyte death. Here, we reported that OA chondrocytes showed a significant increase of cell death rate and the imbalance of cartilage matrix catabolism. We found that the distribution of skeleton protein F-actin was disordered. In addition, the volume-sensitive chloride current of OA chondrocytes decreased significantly with the increase of the expression levels of inflammation-related proteins caspase-1, caspase-3, and NLRP3. Moreover, interleukin-1β (IL-1β) showed a potential to activate the chloride current of normal chondrocytes. These results indicate that IL-1β-induced chloride channel opening in chondrocytes may be closely related to the occurrence of OA. This chloride channel opening process may therefore be a potential target for the treatment of OA.

## Introduction

Osteoarthritis (OA) is the second leading cause of dyskinesia in adults, and about 80% of OA patients experience various degrees of activity restriction. Under pathological conditions, due to long-term weight-bearing and mechanical damage, a large number of inflammatory factors are secreted, which stimulates articular cartilage tissue destruction. Furthermore, inflammatory mediators in the joint can induce the inflammatory necrosis of chondrocytes and trigger abnormal cartilage matrix metabolism. These changes result in detrimental effects on both articular cartilage structure and function [1,2]. In particular, the inflammatory necrosis of chondrocytes plays a very important role in the development of OA.

It has been shown that decreasing levels of extracellular osmotic pressure in turn induce the release of the inflammatory factor interleukin (IL)-1β, followed by the activation of the NLRP3 inflammasome and caspase-1 [3]. Pathological cells in a hypo-osmotic environment will continue to swell until the cell membrane ruptures, leading to the release of cell contents and the triggering of a strong inflammatory response. This may be one of the main forms of chondrocyte death during OA [4,5]. Of course, there are other important mechanisms of OA, such as obesity and adipokines. Obesity causes inflammation of cartilage metabolism, and many of the adipokines are pro-inflammatory factors, such as leptin and adipsin [6,7]. Abnormal mechanical force is also an important factor leading to cartilage loss. Physiological and pathological overload will affect the quality of life of each cartilage cell in the joint. For example, higher dynamic medial knee load, abnormal kinematics and gait will cause abnormal distribution of load forces in the joints. Mechanical factors, such as the rupture or injury of the anterior cruciate ligament and the integrity of the meniscus, will also affect the distribution of load forces in the entire joint [8,9]. These mechanical factors are important causes of OA.

Chloride channels play an important role in the regulation of osteoclast bone resorption, bone mineralization, and bone mass changes [10–12]. Our previous research revealed that estrogen activates ClC-3 chloride channels and promotes bone formation [13,14]. Another study confirmed that, in the hypo-osmotic environment of OA, the death of chondrocytes is accompanied by the downregulation of ClC-7 chloride channels [15]. However, whether there is a relationship between the regulation of chloride channel function driven by inflammatory factors and the occurrence of OA remains unclear.

In this study, we sought to find the mechanism by which inflammatory factors regulate the chloride channels in the setting of cartilage matrix synthesis–catabolic imbalance, which leads to OA.

## Materials and Methods

### Isolation and culture of human chondrocytes

The cartilage joint tissue and chondrocytes used in this study were collected from patients who underwent arthroplasty in the Department of Orthopaedics and Arthrology of the Shenzhen Second People’s Hospital from September 2017 to June 2020. The control group in this study included female patients diagnosed with femoral neck fractures, while the experimental group included female patients with a unilateral or bilateral knee joint diagnosis of OA. Among them, there were seven patients with femoral neck fracture, and their age was 79.28±3.92 years. In the OA group, there were 15 patients with an age of 66.13±2.76 years. Specimens of knee joints and femoral heads were gathered for assessment after surgery. After the operation, the specimens were minced and digested within 6 h. The study was approved by the Ethics Committee of the Shenzhen Second People’s Hospital. In all cases, treatment consent and study inclusion consent forms were signed by the patients before specimens were collected.

After the specimens were collected from the operating room, they were washed with saline three times, and the cartilage was cut into small pieces at 1 mm × 1 mm × 1 mm with a surgical blade. After being washed with physiological saline, the specimen was digested with 1 mg/ml of type II collagenase (Sigma-Aldrich, St Louis, USA) at 37°C for 8–12 h in an incubation bath. After washing and removal of the type 2 collagenase and impurities with a filter, chondrocytes were added to the culture medium (DMEM; ThermoFisher Technology (China) Co., Ltd, Shanghai, China). The complete culture medium for chondrocytes contained 10% fetal bovine serum, 100 IU/ml of penicillin (Sigma-Aldrich, St Louis, USA), 100 µg/ml of streptomycin (Sigma-Aldrich), 1× nonessential amino acid (11-140-050; Gibco, Carlsbad, USA), 1×10^–2^ M HEPES, 2×10^–4^ g/ml L-ascorbic acid, and 4.6×10^–5^ g/ml L-proline. The isolated chondrocytes were routinely cultured in a sterile cell incubator at a constant temperature of 37°C with 5% CO_2_, 95% air, and 100% humidity. First- and second-generation cells were collected for experiments.

### Toluidine blue and propidium iodide staining

After digestion and resuspension, the chondrocytes were planted on slides and paraformaldehyde (4%) was added for fixation for 30 min and then 1% toluidine blue staining solution (Sigma-Aldrich) was added and stained for 2 h, followed by three times wash with PBS. Finally, neutral resin was added for sealing and slides were observed and photographed under a pathological microscope (CX41; Olympus, Tokyo, Japan).

Similarly, in the propidium iodide (PI) staining experiment, chondrocytes on slides were fixed and cleaned, and then PI working solution (Beyotime Biotechnology, Shanghai, China) was added for incubation for 30 min at room temperature. After being washed again, the cells were photographed under a fluorescence microscope (DFC450C; Leica, Wetzlar, Germany).

### Immunofluorescence and chloride ion fluorescent probe staining

Chondrocytes cultured in a confocal dish were collected and washed with PBS. Paraformaldehyde (4%) was added for fixation for 20 min and then cells were washed with PBS. After permeabilization with 0.5% Triton X-100 for 15 min, cells were blocked with 5% BSA for 1 h at room temperature. Then, one or two of the primary antibodies were added and incubated overnight at 4°C in the dark. The primary antibodies used included Cy3-conjugated rabbit anti-collagen I antibody (bs-10423R-Cy3; Bioss, Beijing, China), FITC-conjugated rabbit anti-collagen II antibody (bs-10589R-FITC; Bioss), AF488-conjugated rabbit anti-caspase-1 P10 antibody (bs-0169R-AF488; Bioss), Cy3-conjugated mouse anti-caspase-3 antibody (bsm-33284M-Cy3; Bioss), and anti-NLRP3 monoclonal immunoglobulin G (MAB7578; R&D Systems, Minneapolis, USA). Subsequently, cells were washed with 0.5% BSA and incubated with the corresponding secondary antibody for 2 h when necessary. Then, the cells were washed with PBS and stained with 4ʹ,6-diamidino-2-phenylindole (DAPI) for 5 min. Finally, cells were washed with PBS and images were taken using an oil lens under a confocal microscope (ZEISS LSM 800; Carl Zeiss AG, Oberkochen, Germany).

The digested chondrocytes were seeded on a confocal dish. Cells were cultured overnight and washed twice with PBS. Then isotonic solution and hypotonic solution containing MQAE (10 mM; Beyotime) were added respectively. Cells were put back in the incubator and continued to incubate for 1 h, washed with PBS twice, and then stained with Dil (10 μM; Beyotime) for 10 min. Finally, cells were washed with PBS three times and images were taken under a confocal microscope.

### Histology and immunohistochemistry

Briefly, the cartilage surface was cut with a surgical blade, fixed in 4% paraformaldehyde, and paraffin-embedded in the coronal position. The paraffin blocks were sectioned at a thickness of 5 μm, and the resulting sections were deparaffinized in xylene, hydrated with graded ethanol, and stained with hematoxylin and eosin (Beyotime). Photographs were taken using the CX41 pathological microscope.

### Solutions and chemicals

The solution ingredients are consistent with what we have reported previously [14,16,17]. Extracellular fluids with different osmotic pressures were adopted in the present experiment, including isotonic solutions (Iso) with an osmotic pressure of 300 mOsmol/l and hypotonic solutions (47% Hypo) with an osmotic pressure of 160 mOsmol/l. The isotonic solution contained 70 mM of NaCl, 0.5 mM of MgCl_2_, 2 mM of CaCl_2_, 10 mM of HEPES, and 140 mM of D-mannitol. Relative to the isotonic solution, the hypotonic solution contained all the same components except 140 mM of D-mannitol. The pipette solution consisted of 70 mM of *N*-methyl-D-glucamine Cl^−^, 1.2 mM of MgCl_2_, 10 mM of HEPES, 1 mM of EGTA, 140 mM of D-mannitol, and 2 mM of ATP. The pH values of the extracellular and the internal solution were adjusted to 7.4 and 7.25, respectively. The osmotic pressure was measured with an automatic cryoscopic osmometer (Osmomat030; Gonotec, Berlin, Germany). The chloride channel blocker DIDS (Sigma-Aldrich) was dissolved in dimethyl sulfoxide and freshly diluted to 100 mM working solution before use.

### Patch-clamp current recording

Before the patch-clamp current experiment, cells were re-suspended and seeded on a 22-mm round glass slide and incubated at 37°C for 12 min. The whole-cell chloride current was recorded with a preamplifier (model EPC-7/EPC-10; Heka Elektronik, Lambrecht, Germany) after the micropipette-adsorbed cells had formed a high level of impedance sealing in excess of 1 GΩ at 20–24°C. The cell membrane potential was held at the Cl^−^ equilibrium potential (0 mV) and stepped to 200-ms pulses of 0, ±40, and ±80 mV repeatedly, with 4-s intervals between the pulses. The laboratory interface on a computer (model 1401 or EPC-10 USB; Heka Elektronik) was used to record the voltages and currents with a sampling rate of 3 kHz.

### Statistical analysis

Data were expressed as the mean±SEM. The differences between the two groups were compared using the Student’s *t*-test by SPSS 13.0 software (SPSS, Chicago, USA). *P*<0.05 was considered statistically significant.

## Results

### Cell mortality rate was increased in human OA chondrocytes

**Figure 1.**
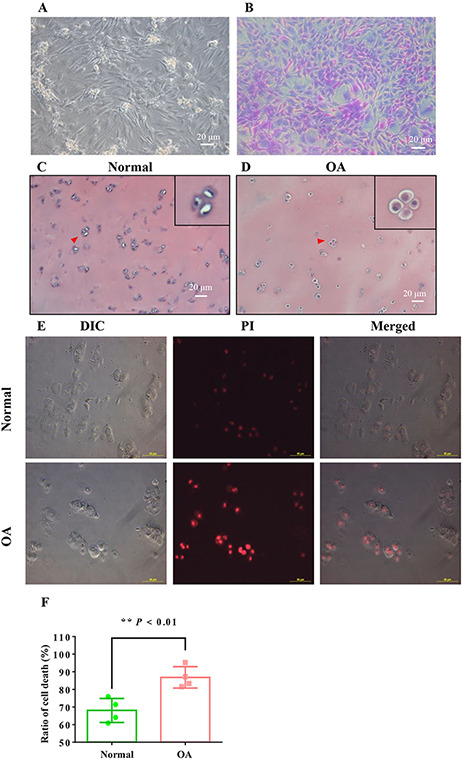
**Cell volume and mortality rate were increased in OA chondrocytes** (A,B) Typical images of normal chondrocytes in a bright field and stained with toluidine blue, respectively. (C,D) Hematoxylin and eosin staining results of human normal or OA chondrocytes, respectively. The nuclei of chondrocytes are blue and the cytoplasm is transparent. (E) The integrity staining of freshly isolated chondrocyte membrane (the dye used was PI). Cells with incomplete membranes are in dark red fluorescent color. (F) The rate of cell death in normal and OA chondrocytes, respectively (*n*=126–155 cells in four views, **P<0.01 vs the normal group).

After 3–5 days of culture, the chondrocytes began to adhere to the walls of culture bottles or Petri dishes and grow. The chondrocytes were round, with two or three cells clustered together or distributed in piles. The chondrocytes often formed triangles or polygons as shown in **Fig. 1A**. Chondrocytes express glycosaminoglycans specifically and can be specifically stained by toluidine blue, showing blue-violet crystals. The cultured cells were stained with toluidine blue for the identification of chondrocytes (**Fig. 1B**).

In addition, small squares on the surface of articular cartilage were embedded in paraffin. The nuclei were stained bright blue by hematoxylin and eosin staining, while the cytoplasm remained transparent. The average size of chondrocytes in the normal group was uniform, and the cell diameter was about 10–20 μm (**Fig. 1C**,**D**).

PI staining was used to observe the cell death of uncultured normal and OA chondrocytes. As shown in **Fig. 1E**, only some of the chondrocytes in the normal group could be lightly stained by PI, with intact membranes. In contrast, most of the OA chondrocytes could be deeply stained by PI, suggesting that the membranes of OA cartilage were incomplete or compromised. We counted 126–155 chondrocytes in two normal and seven OA patients and found that the number of cells stained by PI was also higher than that in the normal group, which was 68.07±3.41 (*n*=126 cells in four views) and 86.83±3.04 (*n*=155 cells in four views), respectively (**Fig. 1E**,**G**; *P*<0.01 vs the normal group).

### Cell matrix loss was increased and the distribution of cytoskeletal protein was strongly disrupted in human OA chondrocytes

**Figure 2.**
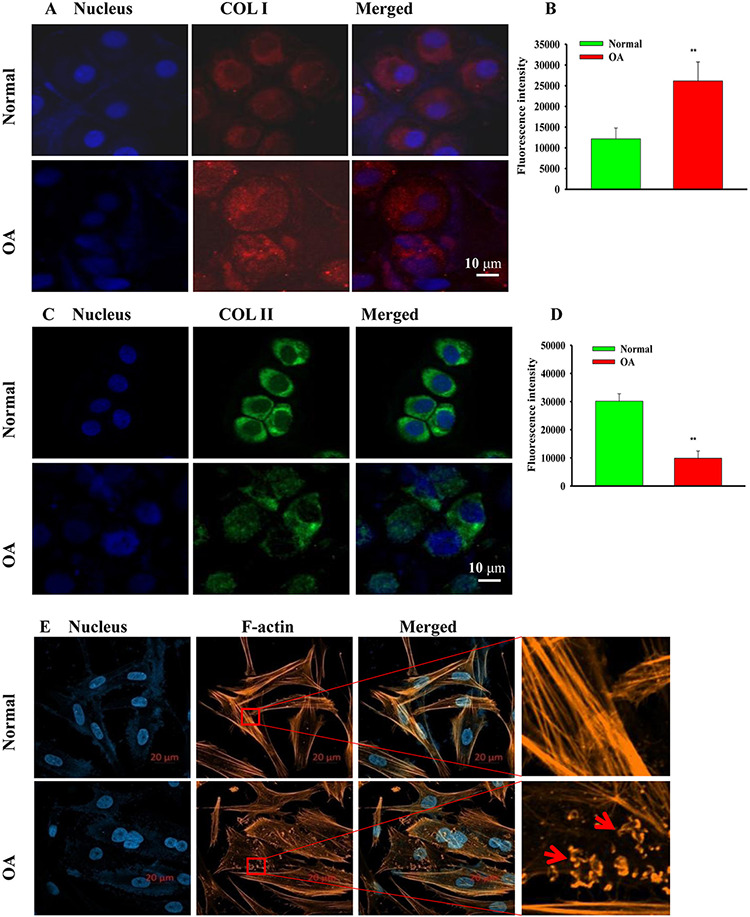
**Expression levels of COL I, COL II, and F-actin** (A) The expression of COL I (red) identified by immunofluorescence in normal and OA chondrocytes. (B) The average fluorescence intensity of COL I (the mean±SE, *n=*3) (**P<0.01). (C) The expression of COL II (green). (D) The average fluorescence intensity of COL II (the mean±SE, *n=*3) (**P<0.01). (E) The difference in F-actin protein expression distribution between the normal group and OA group chondrocytes.

Then, we continued to observe the expressions of COL I and COL II (Collagenase type II) in chondrocytes in the normal and OA groups by immunofluorescence staining. Results showed that the expression of COL I was limited, and the expression of COL II was abundant in normal chondrocytes, indicating that the loss of the extracellular matrix was less severe in the normal group. Meanwhile, in the OA group, the expression of COL I was increased, while that of COL II was reduced (**Fig. 2A**,**C**). The relative fluorescence intensity of the cells also supported these results (**Fig. 2B**,**D**; *P*<0.01 vs the normal group).

The cytoskeleton is a network of protein fibers involved in cell migration, adhesion, morphological changes, and signal transduction. Phalloidine can specifically bind actin. Here, we used rhodamine-labeled phalloidine to observe the difference in chondrocytoskeleton proteins between the normal group and the OA group. It was found that the chondrocytoskeleton in the normal group was arranged in a normal and orderly manner, while that in the OA group was arranged in a disorderly manner, similar to a broken spring. These findings suggest that OA chondrocytoskeleton proteins cannot maintain the normal functions of cells, further aggravating the occurrence of OA (**Fig. 2E**).

### Volume-sensitive chloride current was reduced in human OA chondrocytes

**Figure 3.**
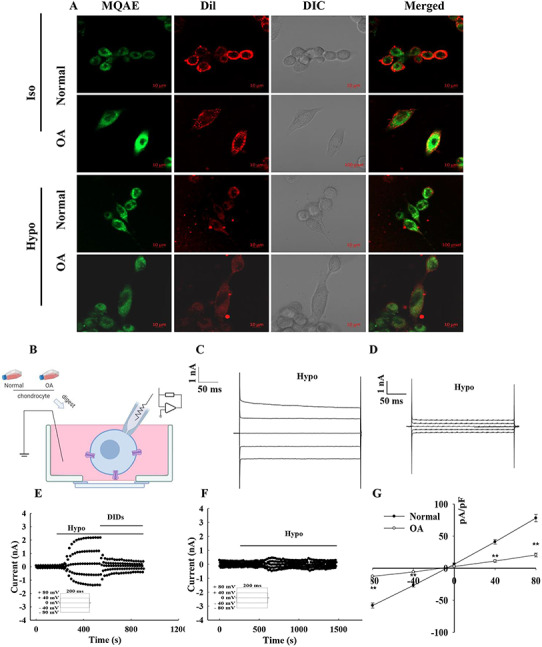
**Whole-cell patch-clamp recording of volume-sensitive chloride currents in normal and OA chondrocytes** (A) The chloride ion fluorescence probe was used to detect the difference of chloride ion in normal and OA cartilage cells. The cultured P1 generation cells were seeded on a confocal dish and cultured overnight. Then isotonic (300 mOsmol/l) and hypotonic (160 mOsmol/l) extracellular fluid containing MQAE (10 mM) were added and incubated for 1 h. Green fluorescence is chloride ion fluorescent probe MQAE, excitation wavelength is 408 nm, false color: green. Red is cell membrane pigment, excitation wavelength is 543 nm, false color: red. (B) The pattern of whole-cell patch-clamp recording the chloride current. (C,D) Typical graphs of transient current under the hypotonic condition within 200 ms in normal or OA chondrocytes. (E,F) The typical time course of the Cl^−^ current activated by hypotonic bath solution in normal or OA chondrocytes. (G) The current–voltage (I–V) relationship recorded in normal or OA chondrocytes.

MQAE was used to detect the difference in chloride ion concentration between normal and OA chondrocytes. Results showed that when the cells were at 300 mOsmol/l, the fluorescence intensity of OA chondrocytes was higher than that of normal chondrocytes, indicating that the chloride ion concentration in OA chondrocytes was lower than that in normal chondrocytes (**Fig. 3A**). When the extracellular osmotic pressure further dropped to 160 mOsmol/l, the fluorescence intensity was just the opposite. At this time, the fluorescence intensity of normal chondrocytes was stronger than that of OA chondrocytes. This change is related to the opening of chloride channels. At 300 mOsmol/l, OA chondrocytes open more chloride channels than normal chondrocytes. However, when the osmotic pressure further drops to 160 mOsmol/l, normal chondrocytes open a large number of chloride channels, while OA chondrocytes can only open a small amount compared with normal chondrocytes. In order to further prove this difference, the whole-cell patch clamp method was used to further record the changes in the chloride current of normal and OA chondrocytes.

We first detected the mRNA expression levels of several chlorine channels that may be volume-sensitive chlorine channels. The expressions of *ClC-3, ClC-7*, and *LRRC8A* were detected in normal and OA chondrocytes, respectively. As shown in **Supplementary Fig. S1**, the mRNA level of *LRRC8A* was significantly increased in OA chondrocytes. Subsequently, the difference in volume-sensitive chloride current during the hypotonic solution (Hypo) activation of normal and OA chondrocytes was recorded by the whole-cell patch-clamp method in the first generation of cultured chondrocytes. **Figure 3B** shows the pattern of the patch-clamp experiment, which recorded the volume-sensitive chloride current in human chondrocytes. As shown in **Fig. 3C**,**D**, the typical traces of Cl^−^ current were recorded in the human chondrocytes under the hypotonic condition. The hypotonic-activated chloride current of chondrocytes in the normal group was significantly higher than that in the OA group. **Figure 3E**,**F** shows the typical time courses of chloride current in the normal and OA groups, respectively. The current density values of normal and OA chondrocytes in isotonic solution were 4.21±0.84 pA/pF at +80 mV and −4.78±0.52 pA/pF at −80 mV (normal) and 5.50±1.19 pA/pF at +80 mV and −5.56±1.08 pA/pF at −80 mV (OA). Meanwhile, perfusion with the hypotonic solution activated the currents with a density of 78.18±5.66 pA/pF at +80 mV, −58.13±4.00 pA/pF at −80 mV (normal), 20.69±2.91 pA/pF at +80 mV, and −12.59±0.42 pA/pF at −80 mV (OA), suggesting that the volume-sensitive chloride current of OA chondrocytes activated by hypotonic solution was significantly decreased when compared with the volume-sensitive chloride current of normal chondrocytes (**Fig. 3G**; *P* < 0.01 vs normal group, *n*=3–6).

### The expression levels of caspase-1 and caspase-3 protein were increased in human OA chondrocytes

**Figure 4.**
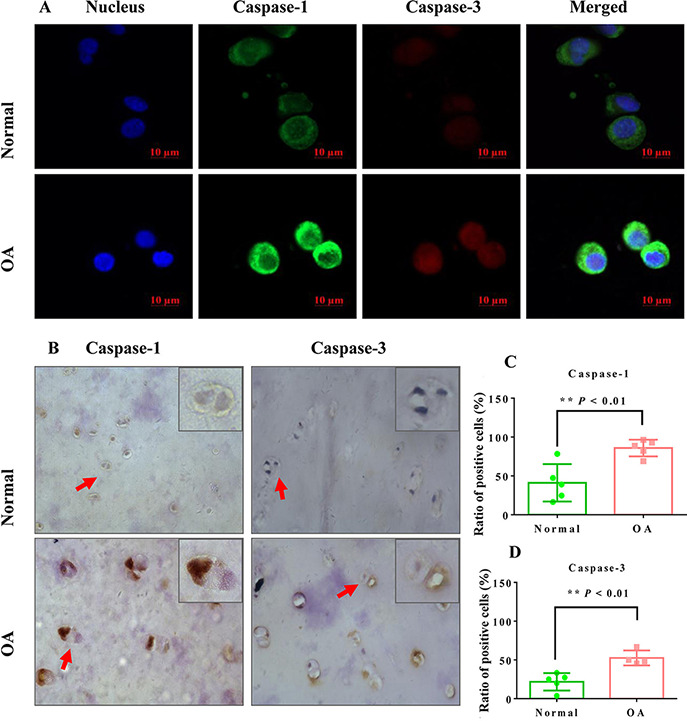
**Expression levels of caspase-1 and caspase-3 detected by immunofluorescence and immunohistochemical staining in normal and OA chondrocytes** (A) The detection of caspase-1 and caspase-3 expressions by immunofluorescence. The digested chondrocytes are directly centrifuged on the slide for staining. The nuclei were stained with DAPI (blue), caspase-1 was labeled by Alexa Fluor 488 (green), and caspase-3 was labeled by Cy3 (red). Fluorescent images were obtained by confocal microscopy. (B) The detection of caspase-1 and caspase-3 expression levels by immunohistochemistry. Typical images were obtained with a pathological microscope. (C,D) The rate of positive cells for caspase-1 and caspase-3 (*n*=339–411 cells in five views, **P<0.01 vs the normal group; *n*=223–269 cells in five views, **P<0.01 vs the normal group).

**Figure 4A** shows that the green fluorescence intensity in OA chondrocytes was significantly increased, suggesting that the expression of caspase-1 in OA chondrocytes was increased. However, although the intensity of red fluorescence in OA chondrocytes was increased, it remained rather low. Meanwhile, the expression of caspase-3 was less than the expression of caspase-1 in OA chondrocytes, suggesting that OA chondrocyte inflammatory necrosis is an important pathway for cell death.

Immunohistochemical results also revealed that the expression levels of caspase-1 and caspase-3 in OA chondrocytes were increased (**Fig. 4B**). Meanwhile, we also determined the positive expression rates of caspase-1 and caspase-3. The results showed that the positive expression rate of caspase-1 in OA chondrocytes was 85.85%±4.79%, which was significantly higher than that of the normal group chondrocytes (41.12%±10.73%) (**Fig. 4C**; *P*<0.01 vs OA group, *n*=339–411 cells in five views). The positive expression rate of caspase-3 in normal chondrocytes was 52.54%±4.80%, which was also higher than that of the normal group chondrocytes (21.85%±5.02%) (**Fig. 4D**; *P*<0.01 vs normal group, *n*=223–269 cells in five views).

### The NLRP3 protein expression level was increased in human OA chondrocytes

**Figure 5.**
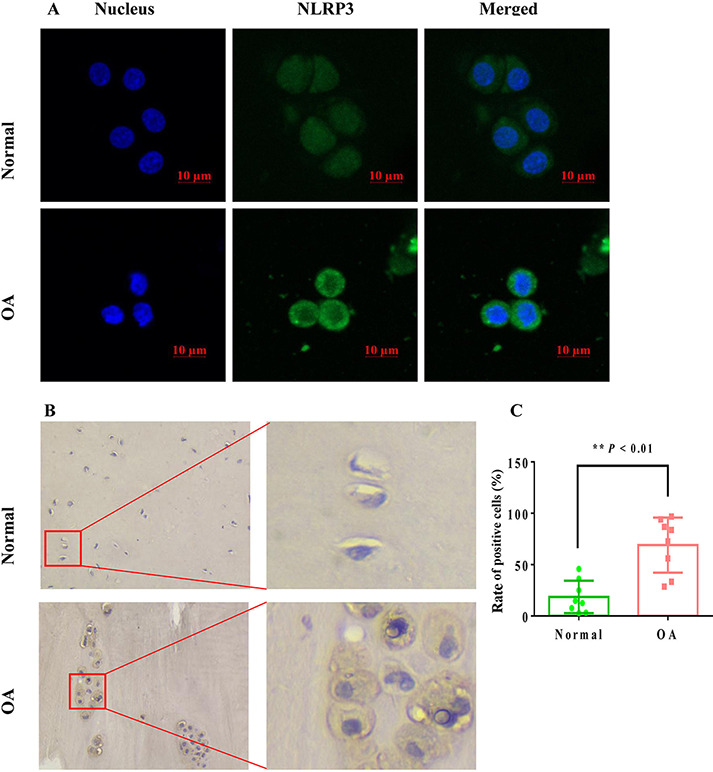
**Expression level of NLRP3 detected by immunofluorescence and immunohistochemical staining in normal and OA chondrocytes** (A) The detection of NLRP3 expression by immunofluorescence. The digested chondrocytes are directly centrifuged on the slide for staining. The nuclei were stained with DAPI (blue), and NLRP3 was labeled by the secondary antibody conjugated with FITC (green). (B) The detection of NLRP3 expression by immunohistochemistry. Typical images were obtained with a pathological microscope. (C) The rate of positive cells for NLRP3 (*n*=198–211 cells in eight views, **P<0.01 vs the normal group).

Subsequently, we observed the difference in the NLRP3 expression level between the normal group chondrocytes and OA group chondrocytes. The green fluorescence of the NLRP3 protein in the normal group was weaker, while the green fluorescence of the NLRP3 protein in the OA group was stronger than that in the normal group (**Fig. 5A**), suggesting that the NLRP3 inflammasome protein level was higher in the OA group than in the normal group, which may be closely correlated with the occurrence of OA. Immunohistochemical results also revealed that the expression level of NLRP3 in the OA group chondrocytes was increased compared with that in the normal group chondrocytes (**Fig. 5B**). Meanwhile, we also determined the positive expression rates of NLRP3. The positive expression rate of NLRP3 of the OA group chondrocytes was 69.02%±9.47%, which was significantly higher than that of the normal group chondrocytes (18.50%±5.60%) (**Fig. 5C**; *P*<0.01 vs OA group, *n*=198–211 cells in eight views).

### IL-1β-induced chloride currents in human chondrocytes

**Figure 6.**
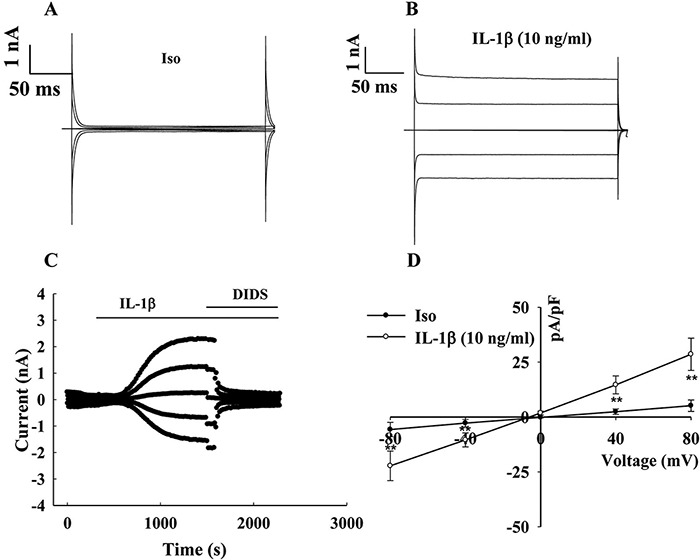
**IL-1β induces chloride currents in chondrocytes** (A,B) The typical graphs of transient current under the conditions of an isotonic solution and isotonic solution containing 10 ng/ml of IL-1β within 200 ms in normal chondrocytes. (C) The typical time course of the Cl^−^ current activated by an isotonic solution containing 10 ng/ml of IL-1β in normal chondrocytes. (D) The current–voltage (I–V) relationship recorded in the context of an isotonic solution and isotonic solution containing IL-1β.

OA chondrocytes highly express the NLRP3 inflammasome and caspase-1 protein and release a large amount of IL-1β. Thus, one question of concern is whether these actions have an effect on chondrocyte chloride channels and participate in the regulation of the inflammatory death of chondrocytes. To address this question, we used the whole-cell patch-clamp technique to record the chloride current of normal chondrocytes. Similarly, cells were first incubated in isotonic control bath solution, after the current was stabilized, isotonic solution containing IL-1β (10 ng/ml) was replaced for infusion. The current density values of the isotonic and isotonic solutions containing IL-1β were 5.17±2.59 pA/pF at +80 mV, −5.69±3.27 pA/pF at −80 mV (Iso), 28.58±7.35 pA/pF at +80 mV, and −22.25±6.72 pA/pF at −80 mV (IL-1β), respectively, suggesting that IL-1β significantly activates the chloride channels in chondrocytes, which may be a potential important signal involved in OA (**Fig. 6**; *P* < 0.01 vs Iso, *n*=6).

## Discussion

In this study, we investigated the biological effects of IL-1β on the characteristics of chondrocytes, focusing on the chloride channels in OA patients and normal controls. We found that the main fibrotic chondrocytes in OA patients displayed the following characteristics: increased inflammatory necrosis, severe COL II loss, and cytoskeleton disorder. Meanwhile, OA chondrocytes highly expressed caspase-1, caspase-3, as well as NLRP3 proteins. There are volume-sensitive chloride channels in chondrocytes, and inflammatory factor IL-1β can induce the opening of chloride channels in normal chondrocytes. IL-1β-induced chloride channel opening may have an important association with the development of OA. Retaining the cartilage matrix–metabolic balance is the physiological basis for maintaining normal articular cartilage function [18]. Here, we observed that COLI and COLII are out of balance in OA chondrocytes. We also found that, under pathological conditions, in the setting of the combined action of a large number of inflammatory factors and mechanical stimulation, the expression levels of MMP-13 and ADAMTS5 were increased, and the catabolism of articular cartilage matrix was significantly greater than anabolism, resulting in the degeneration of articular cartilage seen in OA [19,20]. In a previous study, we found that estrogen can target the expression of MMP-13 through microRNA-140, which may be an important reason why postmenopausal women suffer from OA at significantly higher rates than men [21].

In this study, we also observed a severe imbalance in cartilage matrix synthesis and catabolism and increased rates of cytoskeletal protein-breaking cell death. These findings are closely related to the significantly increased expression levels of caspase-1, caspase-3, as well as NLRP3. Previous studies have suggested that the activation of NLRP3 is typical of pyroptosis [22,23]. When NLRP3 is activated, caspase-1 is further activated, thereby releasing IL-1 [24]. Pyroptosis is characterized by the continuous expansion of cell volume until the cell membrane ruptures, a phenomenon similar to the obvious changes observed among OA chondrocytes [25,26].

Changes in cell volume are important factors that induce inflammation [27]. It has been demonstrated that hypotonicity induces cell swelling, which, in turn, activates the release of caspase-1 and IL-1β through the NLRP3 inflammasome [28]. Uric acid crystals can activate the NLRP3 inflammasome and trigger IL-1β secretion, suggesting that inflammasome activation and IL-1β secretion are the driving factors behind the occurrence of gout [29]. We noted that the osmotic pressure in the normal joint cavity is 400 mOsmol/l, while the osmotic pressure in the joints of patients with OA is significantly reduced [30,31]. Low osmotic pressure may be an important factor in the continuous occurrence of inflammation in the joint cavity.

**Figure 7.**
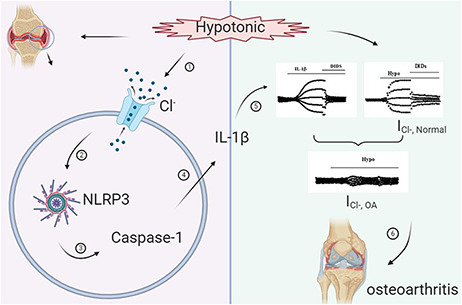
**The potential relationship between IL-1β-induced chondrocyte chloride current and OA** The persistent hypotonic environment in joint fluid induces chondrocyte volume increase and Cl^−^ ion outflow. At the same time, it activates the NLRP3 inflammasome and then activates caspase-1, resulting in the release of IL-1β. IL-1β then activates the chloride channels of chondrocytes and continuously induces their opening, which leads to a pathological decrease in the chloride current. This may have a potential relationship with the occurrence of OA.

Chloride ion channels play a role in regulating the occurrence of OA [32]. Previous studies have pointed out that ClC-3 messenger expression exists in the articular cartilage [33]. In addition, in human tissues, the presence of ClC-3, ClC-5, and ClC-7 can also be detected [15,34,35]. These chloride channels are actively involved in regulating the function of chondrocytes. The joint cavity is continuously hypotonic, resulting in the downregulation of ClC-7 chloride channels [15]. Chloride channel function declines, far before the loss of the chondrocyte tissue matrix [31]. Our results showed that the volume-sensitive chloride current of OA chondrocytes is significantly reduced, which may be linked to the continuous hypotonic state of OA chondrocytes, resulting in the downregulation of chloride channels. In addition, short-term hypotonic stress or the inhibition of ClC-7 expression can also cause chondrocyte death. Chloride channels are closely related to apoptosis and cell volume regulation, but their molecular types are difficult to figure out. The involvement of several genes has been proposed, including *LRRC8A, ClC-3*, and *VRAC* [36–39]. Nonetheless, the involvement of chloride channels in the regulation of the biological function of chondrocytes is well-established (**Fig. 7**).

Hypo-osmosis is an extracellular red flag for inflammation. Hypo-osmosis can induce increased IL-1β-dependent release of inflammatory factors. The release of inflammatory factors induced by hypo-osmosis is associated with activated NLRP3 inflammasomes [22]. It has been shown that Cl^−^ is involved in mediating the activation and release of IL-1β in NLRP3 inflammasomes [40]. In addition, hypotonia is known to activate volume-sensitive chloride channels, and the hypotonic-induced release of IL-1β further promotes the opening of chloride channels and aggravates the inflammatory state. Here, we tried to add IL-1β outside the cell to observe its effect on chloride current and found that low concentrations of IL-1β activated chloride currents in normal chondrocytes, suggesting that IL-1β-induced chloride channel opening may potentially be associated with chondrocytes in a manner pointing toward OA. Nevertheless, we did not further assess the molecular identity of IL-1β-induced chloride channels. We speculate that this might be related to the volume-sensitive chloride channel LRRC8A that was recently reported [36,37], but further work is needed to verify this speculation.

## Supplementary Material

gmab010_Supp
