# Supplementary figures and images for: Interleukin 1 beta-induced chloride currents are important in osteoarthritis onset: an in vitro study
Source: Acta Biochim Biophys Sin (Shanghai). 2021 Mar 2;53(4):400–9. doi: 10.1093/abbs/gmab010 (PMC7996641; doi:10.1093/abbs/gmab010)

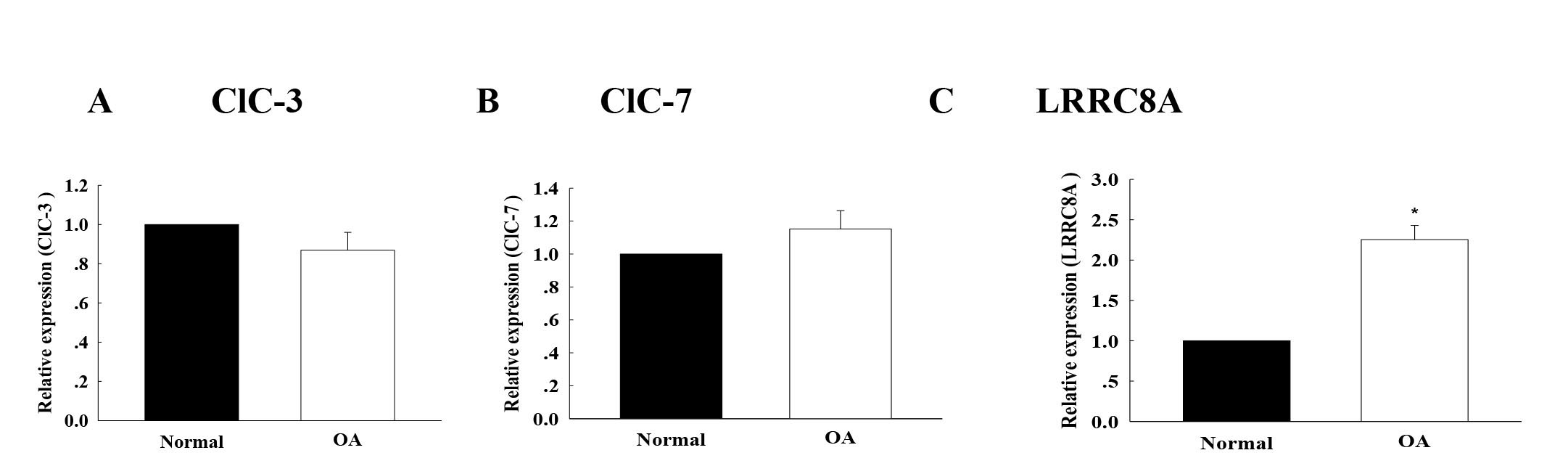

Supplement: gmab010_Supp [file gmab010_supp.zip › 20447Fig._S1.jpg]
